# Association between long-term air pollution exposure and COVID-19 mortality in Latin America

**DOI:** 10.1371/journal.pone.0280355

**Published:** 2023-01-17

**Authors:** Jorge A. Bonilla, Alejandro Lopez-Feldman, Paula C. Pereda, Nathaly M. Rivera, J. Cristobal Ruiz-Tagle

**Affiliations:** 1 Department of Economics, Universidad de Los Andes, Bogota, Colombia; 2 Environment for Development, University of Gothenburg, Göteborg, Sweden; 3 Department of Economics, Centro de Investigacion y Docencia Economicas, Mexico City, Mexico; 4 Department of Economics, University of São Paulo, São Paulo, Brazil; 5 Department of Economics, University of Chile, Santiago, Chile; 6 Department of Geography & Environment, London School of Economics and Political Science, London, United Kingdom; Universidad Nacional Autonoma de Nicaragua Leon, NICARAGUA

## Abstract

Recent studies have shown a relationship between air pollution and increased vulnerability and mortality due to COVID-19. Most of these studies have looked at developed countries. This study examines the relationship between long-term exposure to air pollution and COVID-19-related deaths in four countries of Latin America that have been highly affected by the pandemic: Brazil, Chile, Colombia, and Mexico. Our results suggest that an increase in long-term exposure of 1 *μ*g/m^3^ of fine particles is associated with a 2.7 percent increase in the COVID-19 mortality rate. This relationship is found primarily in municipalities of metropolitan areas, where urban air pollution sources dominate, and air quality guidelines are usually exceeded. By focusing the analysis on Latin America, we provide a first glimpse on the role of air pollution as a risk factor for COVID-19 mortality within a context characterized by weak environmental institutions, limited health care capacity and high levels of inequality.

## Introduction

Ambient air pollution poses a significant threat to human health as it is linked to millions of deaths every year [1, 2]. Previous studies show that exposure to poor air quality is associated with lower respiratory infections, chronic obstructive pulmonary disease, ischemic heart disease, and strokes, among others [3–6]. By weakening the immune system, long-term exposure to air pollution may also affect the physiological response to new diseases, such as The New Coronavirus Disease (COVID-19) (e.g., [7, 8]). In this paper, we explore this link by looking at long-term air pollution exposure and deadly outcomes from COVID-19 in the context of Latin America.

Countries in this region often lack strong institutions to successfully control and promote air pollution abatement. Unsurprisingly, air pollution has become the biggest environmental hazard in Latin America [9], where only four cities, out of fifty-seven that monitor fine particulate matter (PM_2.5_) concentrations, meet World Health Organization (WHO)’s yearly guidelines of 10*μ*g/m^3^ [10]. Indeed, recent PM_2.5_ pollution records in Bogota, Mexico City, Santiago, and Sao Paulo, show concentrations are more than six times above WHO’s recommended standard (see S1 Fig). This leaves more than 33 million people in the region at risk.

The costs of exposure to air pollution may substantially increase when coupled with weak environmental institutions [11] and limited health care capacity [12]. Indeed, Latin America is characterized by health care systems with limited and unequal capacity [13] and an important fraction of its population lives in poverty. All these further exacerbates the adverse effects of poor air quality [14, 15].

Latin America has been severely affected by the coronavirus pandemic. Health systems throughout the region reached critical levels and hospitals became short of capacity during peak periods. Several countries followed a set of stringent policies to address the spread of coronavirus, with many cities imposing lockdowns and stay-at-home orders sending millions of people to confinement. Despite these efforts, the region experienced one of the highest mortality rates in the world. In fact, whereas Latin America’s total population represents only 8.4 percent of the global population, the total number of deaths from COVID-19 stands at roughly 1.3 million [16], which is equivalent to almost 30 percent of the fatalities worldwide. The extent to which these deadly outcomes have been affected by the historically elevated levels of air pollution in the region remains yet unknown.

In this paper we examine the relationship between long-term air pollution exposure and deadly outcomes from COVID-19 in Latin America by focusing on Brazil, Chile, Colombia, and Mexico, countries with some of the highest mortality rates in the region and that have experienced more than 1 million fatalities by January 2022. The analysis relies on municipality-level satellite data of average PM_2.5_ concentrations from 2000 to 2018. We combine this data with official statistics on municipal-level COVID-19 confirmed fatalities during 2020. Throughout our estimations, we account for socioeconomic and health characteristics, such as the spatial variation in the timing of the outbreak and time of exposure to the virus, as well as potential country- and state-specific unobservable effects that may, otherwise, act as confounders.

Our results show a positive and significant statistical association between long-term PM_2.5_ concentrations and COVID-19 mortality in both a pooled sample as well as in country-specific analyses. This relationship turns stronger for municipalities that are located in metropolitan areas, which usually exceed the WHO air quality guidelines. Our findings are consistent with the notion that particle air pollution is linked to increased vulnerability, severe health outcomes, and fatal events due to COVID-19 [7, 8, 17–21].

## Related literature

While several studies provide evidence of a statistical association between long-term air pollution and COVID-19 mortality (see [22, 23] for literature reviews), most of them use data from developed countries [24–31]. For example, a positive association has been found for the United States [21], as well as for the Netherlands [19], Italy [18], Spain [32], and Germany [33]. Similar studies explore the link between COVID-19 fatalities and other air pollutants [20, 34–36].

Although the World Meteorological Association (WMT) has called for caution on the interpretation of meteorological and air quality factors as causally affecting COVID-19 spread and mortality [37], there is emerging evidence of the causal links between exposure to air pollution and COVID-19 mortality. For example, evidence of a causal relationship between contemporaneous exposure to PM_2.5_ pollution and COVID-19 mortality has been provided for the United States [38], and a causal relationship between day-to-day variation in pollution by particulate matter with a diameter less than or equal to 10 *μ*m (PM_10_) and COVID- 19 cases and mortality among the elderly has been documented in Germany [39]. Similarly, it has been shown that a rollback of enforcement of environmental regulation by the US-EPA increased PM_2.5_ pollution and COVID-19 cases and deaths [40]. However, more evidence is needed before we can generalize these findings to other regional settings, particularly in the context of developing and emerging economies where air pollution concentrations are critical, COVID-19 spread and mortality are high, and healthcare systems are deficient.

Among the studies for developing and emerging economies, a positive association between long-term exposure to PM_2.5_, PM_10_, and Nitrogen Dioxides (NO_2_), and the risk and severity of COVID-19 infection has been found in China [41]. Meanwhile, districts of India severely affected by PM_2.5_ concentrations experience significant increases in COVID-19 deaths and fatality rates [42], whereas similar evidence has been documented for the metropolitan area of Mexico City [17]. Other studies have explored the relationship between long-term exposure to pollution and the spread of coronavirus in Latin America [43], and looked at air pollution exposure and COVID-19 mortality in Colombia [44].

We add to this literature by exploring the link between exposure to long-term air pollution and COVID-19 mortality for a comprehensive set of countries in Latin America spanning different geographies within the region: northern (Mexico), southern (Chile), central (Colombia), and eastern (Brazil) Latin America. While geographically scattered, these countries share similar characteristics and face common socioeconomic and environmental challenges. In addition, we examine country-specific effects, and effects for both urban and rural localities. By taking advantage of very rich datasets for each country, we also control for a large set of factors that may otherwise confound the relationship between exposure to long-term air pollution and COVID-19 mortality.

Moreover, unlike other developing and emerging economies, countries in Latin America still face one of the highest levels of income inequality in the world, which poses additional challenges to taking on the threat of both air pollution and COVID-19 equally across the region. By focusing the analysis on Latin America, we provide a first glimpse of the role of air pollution as a risk factor for COVID-19 mortality within such a context.

## Materials and methods

### Data

#### COVID-19 mortality

Data on mortality comes from each country’s official statistics. The National Institute of Health and the Ministry of Health, for Colombia and Mexico, respectively, register individual-level deaths per day. In Brazil, individual-level data comes from the Ministry of Health’s surveillance system for Severe Acute Respiratory Syndrome Coronavirus 2 (SARS-CoV-2) cases. For Chile, however, patient-level information is not publicly available. Through the Ministry of Science, the Ministry of Health releases the number of confirmed COVID-19 fatalities, although aggregated at the municipality level. Thus, for consistency of our analysis, we aggregate individual data per municipality for Brazil, Colombia, and Mexico. The data that we use is official data that has been validated by the authorities from each country and is, therefore, the best publicly available data. Moreover, in all four countries under analysis, records of mortality are linked to the patient’s city of residence instead of the city of their decease, which reduces concerns about a potential misclassification bias that may emerge from an incorrect classification of patients across cities. Our final measure of mortality is the total number of COVID-19-confirmed deaths per municipality in each country during 2020.

#### Long-term air pollution concentrations from satellite imagery

Uniform and comprehensive sources of air quality across countries are rare. Ground-level monitoring stations are generally available only for large cities in each country, and their coverage over time generally differs between them. This situation prevents us from using air concentrations from monitoring stations. Instead, we employ satellite imagery as a measure of particle air pollution. Although satellite-based information may be subject to criticism because of its wide spatial resolution, it has the advantage of allowing us access to homogeneous records of air pollution concentrations for all municipalities in our analysis and across several years. We obtain long-term average annual concentrations of fine particulate matter (PM_2.5_) from [45]. This data is available at 0.01 *×* 0.01 degrees (*≈* 1.1 *×* 1.1 km) from 1998 to 2018. From this dataset, we create a long-term municipality-averaged measure of air pollution by computing the mean of PM_2.5_ for grid cells within each municipality and year, from 2000 to 2018. We also compute PM_2.5_ averages for the period 2010–2018 to be used later in a robustness analysis.

#### Covariates

We merge death counts per municipality with PM_2.5_ data and other municipality-level variables in each country. We include several covariates in our estimations to account for possible confounders that may also affect mortality. By doing so, we get an estimate of the link between air pollution and COVID-19 deaths that is statistically isolated from the influence of other factors. These variables account for demographics, socioeconomic characteristics, and health conditions. Although ideally, we would like to consider the same covariates for the four countries, this is not possible due to data availability. Therefore, throughout all our estimations, we use two groups of variables: (i) a common set of covariates available for the four countries (hereafter called the “common-set”); and (ii) a richer set of covariates that, in addition to those variables included in (i), includes variables available for each country (hereafter called the “richer-set”).

*Common-set of covariates*. It comprises a set of eight common covariates available for the four countries. It includes the proportion of people in different age ranges (15–44, 45–64, above 65), to consider the possibility that COVID-19 deaths may concentrate in the older groups of the population. Population density (number of inhabitants per square kilometer in each municipality) is included to allow for differences in the probability of transmission across municipalities. Socioeconomic variables include the proportion of adults that did not finish high school and the proportion of inhabitants that live in rurality. These variables aim to allow for variation in development levels across municipalities. Finally, we include two health-related variables: the number of hospital beds, and the number of days since the first COVID-19 case was detected in each municipality. The pandemic strongly challenged the operation and capacity of existing healthcare systems. An overly stressed healthcare system may affect the timing in which a COVID-19 patient receives medical treatment, and thus increase the risk of death. The number of hospital beds, therefore, informs us of the healthcare capacity available, and indirectly on the preparedness of healthcare systems to face this unprecedented crisis. Lastly, the number of days since the first COVID-19 case informs us about variation across municipalities in the length of exposure to the SARS-CoV-2 virus as well as allowing us to take into account spatial variations in the timing of outbreaks.

*Richer-set of covariates*. It includes the common set of covariates as well as country-specific variables that may affect the risk of COVID-19-related death. The additional co- variates are poverty rate, the Gini coefficient, the proportion of minority and non-minority population, the proportion of people with pre-existing conditions (such as diabetes, hypertension, obesity, and respiratory diseases), the proportion of overcrowding, and access to health services, among others (see S1 Appendix for the complete list of covariates for each country).

### Methods

Our study examines the relationship between long-term air pollution exposure and the COVID-19 mortality rate in a selected group of Latin American countries. Long-term or chronic air pollution exposure aims to reflect exposure throughout an individual’s entire life, or over a long period, where ‘long’ is broadly defined. In this paper, we proxy lifetime exposure as air pollution concentration averaged over the period 2000–2018. Similar to [21], we estimate a linear specification of the relationship between the number of deaths and long-term ambient PM_2.5_ concentrations. To do so, we use a pooled cross-section model of municipalities for four countries: Brazil, Chile, Colombia, and Mexico. We estimate the following equation:

$$\ln\;E[{Deaths}_{ij}]=\beta_{0}+\beta_{1}{Pollution}_{ij}+X_{ij}\gamma +{Country}_{j}+\ln\left({Population}_{ij}\right)+\epsilon_{ij},$$ (1)

where *Deaths*_*ij*_ refers to the cumulative number of deaths in municipality *i* in country *j* during 2020; *Pollution*_*ij*_ is the 2000–2018 satellite-based average of PM_2.5_ concentrations in municipality *i* in country *j*; *Country*_*j*_ is a fixed effect by country; *Population*_*ij*_ is municipality *i*’s population; and *ϵ*_*ij*_ is an error term. The vector **X**_**ij**_ includes a set of covariates that affect the risk of mortality and are available for the four countries at the municipality level (see the common-set of covariates in the data section).

In addition to the pooled Eq (1), we also conduct estimations separately for each country. By doing this, we estimate heterogeneous coefficients regarding the association between air pollution and COVID-19 mortality rates across countries. Furthermore, apart from the covariates used in Eq (1), we include variables that are only available to some countries (see the richer-set of covariates in the data section). The equation to estimate is as follows:

$${\ln\;E[Deaths}_{i}^{j}]=\beta_{0}^{j}+\beta_{1}^{j}{Pollution}_{i}^{j}+Z_{i}^{j}\gamma^{j}+{State}_{i}+\ln\;{\ln\left({Population}_{i}\right)}^{j}+\epsilon_{i}^{j},$$ (2)

where the elements of the equation are defined as above, except for *State*_*i*_ that now represents a state-level fixed effect, and the vector **Z**_**i**_^*j*^ that includes the common-set of variables as well as country-specific covariates (i.e., the richer-set). In the case of Colombia, when the sample is restricted to metropolitan areas the low number of municipalities prevents the use of state-fixed effects in the regressions. Instead, we include a fixed effect for Andean cities. Cities in high altitude tend to share similar socioeconomic and development dynamics, for instance, they are closer to coffee regions.

Our interest in Eqs (1) and (2) is to estimate *β*_1_ and *β*^*j*^, which would reflect the statistical effect of long-term air pollution as a predictor of the number of COVID-19 fatalities in each municipality *i*, for the pooled sample and in each country, respectively. We estimate Eqs (1) and (2) using a Quasi-Maximum Likelihood Poisson Estimator (QMLPE). Compared to the Negative Binomial Maximum Likelihood Estimator (NBMLE), the QMLPE has the advantage of being consistent regardless of the form of the variance. Conversely, if the variance is misspecified, the NBMLE is not consistent [46]. Given that we do not know the correct form of the variance, we use the QMLPE instead of the NBMLE. As a robustness check, we also estimated Eqs (1) and (2) under the assumption of a negative binomial distribution. As S5 and S6 Tables show, results are qualitatively similar to those obtained using a QMLPE. In all regressions we restrict the coefficient of the population size to one, this allows us to estimate the relationship between changes in pollution exposure and mortality rates instead of the number of fatalities. We cluster standard errors at the state level to account for potential correlation across states in each country. Based on the estimates for *β*_1_ and *β*^*j*^, we report incidence rate ratios (IRR), which allows us to interpret estimated changes in mortality as the relative risk of being exposed to increased pollution. Regressions were estimated using the statistical software Stata® 16. Maps were created in ArcMAP® 10.3. An ethics consent was waived for our study after consultation with the IRB of the London School of Economics. Reference number: 116638.

## Results

In the first subsection we present the descriptive statistics of the main data used in the estimation. We then show results for the relationship between long-term PM_2.5_ pollution exposure and COVID-19 mortality rates in Latin America by pooling all municipalities together. Then, in the final subsection, we allow for a heterogeneous relationship between pollution exposure and deaths by estimating country-specific results.

### Descriptive statistics

We present descriptive statistics on the number of COVID-19 fatalities and mortality rates in Table 1 for the countries in our sample. The total number of deaths during 2020 differs in order of magnitude across countries. Fatalities were roughly 17,000 in Chile and 44,000 in Colombia, while they reached 142,000 in Mexico and almost 194,000 in Brazil. Panel A of Table 1 shows that municipality-level average fatality ranges between 35 and 62 deaths across countries. Moreover, Panel B shows that Colombia and Mexico present the lowest and highest average municipality-level mortality rate, with 0.39 and 0.62 deaths per 1,000 people, respectively. Standard deviations indicate variation in fatalities across municipalities within each country, with some experiencing no deaths during 2020.

**Table 1:** Descriptive statistics on 2020 COVID-19 mortality by country.

Country	Mean	Std. Dev.	Min.	Max.	Obs.
*Panel A*. *Number of Deaths*					
Brazil	34.925	326.204	0	15,679	5,550
Chile	49.606	103.743	0	811	345
Colombia	39.402	335.883	0	9,960	1,119
Mexico	61.933	254.58	0	3,903	2,297
*Panel B*. *Mortality Rate*					
Brazil	0.609	0.464	0	3.309	5,550
Chile	0.581	0.514	0	2.495	345
Colombia	0.385	0.359	0	2.299	1,119
Mexico	0.620	0.617	0	11.091	2,297

**Notes:** This table shows the main descriptive statistics on the 2020 COVID-19 number of deaths and mortality rate by country. Observations are at the municipality level. Panel A shows the number of COVID- 19 deaths averaged across municipalities from January 1st to December 31st, 2020. Panel B shows the COVID-19 mortality rate (per 1,000 people) during 2020. Data were retrieved in January 2021.

**Fig 1:**
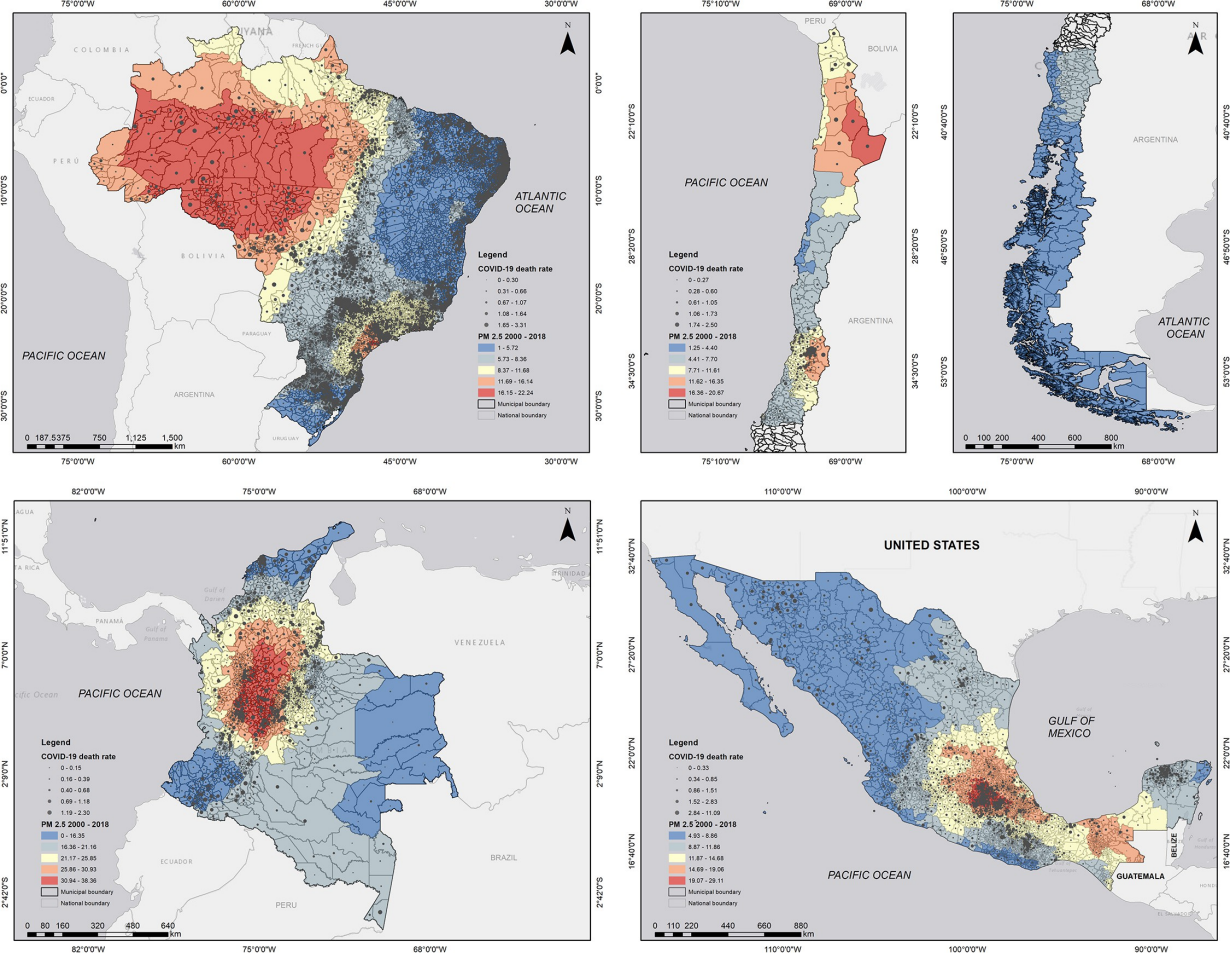
Distribution of long-term pollution (PM_2.5_) concentrations and COVID-19 deaths in Latin America. a: Brazil; b: Chile; c: Colombia; d: Mexico. Notes: This figure shows the spatial distribution of long-term fine particulate matter (PM2.5) concentrations, averaged from 2000 to 2018, and 2020 COVID-19 mortality rate per 1,000 people across municipalities of selected countries in Latin America. We measure pollution in *μ*g/m3, and the data correspond to long-term trends of PM2.5 concentrations obtained from [45]. Data on municipality-level COVID-19 mortality rates come from each country’s official sources for 2020. Maps for this study were created in ArcMap 10.3. The basemaps were adapted from Esri, DeLorme, HERE [47] and have been republished under a CC BY license, with permission, original copyright (2021). The shapefiles for Brazil and Chile were adapted from [48, 49], respectively. The shapefiles for Colombia were adapted from IGAC [50] and have been republished under a CC BY license, with permission, original copyright (2022). The shapefiles for Mexico were adapted from INEGI [51] and have been republished under a CC BY license, with permission, original copyright (2010).

When it comes to the pollution data, country maps of PM_2.5_ levels and mortality rates at the municipal level are shown in Fig 1.

Table 2 displays descriptive statistics of fine particle concentrations averaged from 2000 to 2018 (averages for 2010–2018 are shown in S1 Table). We can observe that while several municipalities exceed the annual WHO Air Quality Guideline of 10 *μ*g/m^3^, there is also a substantial variation in air pollution within and across countries. For instance, some municipalities in Colombia exhibit maximum annual average PM_2.5_ concentrations that are above 38 *μ*g/m^3^, while some Chilean municipalities display average concentrations as low as 1.25 *μ*g/m^3^. Average pollution exposure is the highest in Colombia, while Brazil exhibits the lowest level. These statistics remain practically unchanged when we only consider the period 2010–2018 (see S1 Table), which suggests the lack of substantial air quality improvements in these countries over time.

**Table 2:** Descriptive statistics on 2000–2018 fine particulate matter (PM_2.5_) concentrations.

Country	Mean	Std. Dev	Min.	Max.	Obs.
Brazil	7.39	3.37	3.21	22.24	5,547
Chile	8.23	4.91	1.25	20.67	345
Colombia	23.18	6.51	8.56	38.36	1,119
Mexico	12.96	4.33	4.93	29.11	2,297

**Notes:** This table shows main descriptive statistics on average annual PM2.5 pollution concentrations by country. Pollution is measured in μg/m3. Observations are at the municipality level. Annual concentrations averaged from 2000 to 2018. Data correspond to long-term trends of fine particulate matter concentrations obtained from [45].

**Fig 2:**
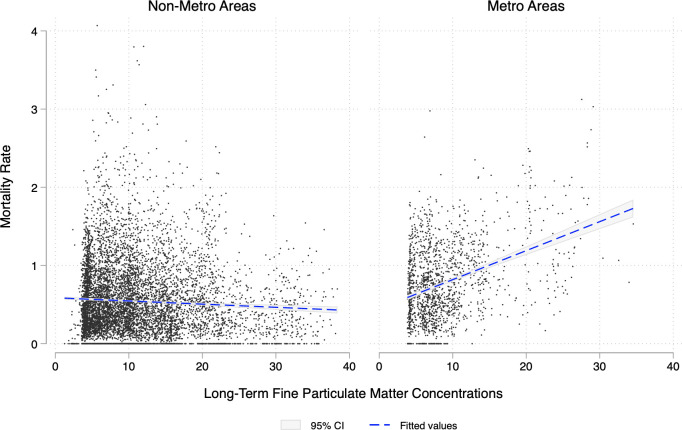
COVID-19 mortality rate and long-term PM_2.5_ concentrations in Latin American municipalities. **Notes:** This figure shows the relationship between COVID-19 mortality rate and long-term average PM2.5 concentrations across Latin American municipalities within metropolitan and non-metropolitan areas. The dashed blue line represents a linear prediction with confidence intervals obtained at the 95% significance (shaded areas). The mortality rate is per 1,000 people. Pollution is measured in μg/m3. Data on mortality rates come from the sources mentioned in the data section. Data on pollution concentrations are long-term trends of fine particulate matter concentrations obtained from [45].

To get a better sense of the link between COVID-19 fatalities and long-term air pollution, Fig 2 depicts the relationship between mortality rate and the satellite-based long-term average PM2.5 concentrations using the entire sample of municipalities across all four countries. We split this relation into metropolitan (right-hand side) and non-metropolitan (left-hand side) areas, according to each country’s classification (see S1 Appendix for more details). For a simple graphical exposition, we overlay a linear prediction plot and its 95%-significance confidence interval. Whereas there seems to be no clear relationship between mortality rate and long-term annual PM2.5 concentrations in municipalities of non-metropolitan areas, we observe that for municipalities within metropolitan areas, there is a positive correlation be- tween these two variables. Due to this contrast, we distinguish between metro and non-metro areas in all our estimations.

### Pooled sample

Table 3 presents parameter estimates for the relationship between long-term exposure to PM_2.5_ and COVID-19 mortality rates across Latin American municipalities. Panel A shows the result using the full sample of municipalities, while Panel B and C show estimation results for municipalities within metropolitan and non-metropolitan areas, respectively. For each set of municipalities, columns exhibit estimation results providing alternative ways of including additional covariates; column (3) represents the specification with the largest available set of controls.

**Table 3:** Long-term average PM_2.5_ exposure and COVID-19 mortality rate in Latin American municipalities.

	(1)	(2)	(3)
*Panel A*. *All Municipalities*			
PM2.5	1.016**	1.004	1.008
	[1.002, 1.030]	[0.995, 1.013]	[0.996, 1.020]
Obs.	9,235	9,235	9,235
*Panel B*. *Metropolitan Areas*			
PM2.5	1.019***	1.015***	1.027***
	[1.007, 1.032]	[1.004, 1.026]	[1.014, 1.040]
Obs.	1,587	1,587	1,587
*Panel C*. *Non-Metropolitan Areas*			
PM2.5	0.998	1.005	1.001
	[0.984, 1.011]	[0.995, 1.014]	[0.988, 1.013]
Obs.	7,648	7,648	7,648
Common-Set of Controls Country Fixed Effects		*×*	*×*
			*×*

**Notes:** This table shows regression estimates of COVID-19 mortality rates on annual PM2.5 concentrations averaged from 2000 to 2018. Estimates shown are incidence rate ratios from Poisson regressions offsetting by population and clustering standard errors at the state level. Observations are municipalities. Common-set includes explanatory variables as defined in the data section. Brackets show 95% confidence intervals. Significance levels: *p < 0.10, **p < 0.05, ***p < 0.01.

Starting with Panel A of Table 3, the results in column (1) indicate a strong and positive relationship between long-term exposure to PM_2.5_ and fatalities associated with COVID-19. In particular, we observe that one additional microgram per cubic meter of PM_2.5_ is associated with a 1.6 percent increase in the mortality rate associated with the coronavirus disease. This effect remains positive, although it lacks statistical significance after controlling for additional factors that may also affect our outcome variable (column (2)), and when unobservable factors common to each country are taken into account (column (3)). Notwithstanding, this result holds positive and turns statistically significant when we look at this relationship within Latin American municipalities that belong to metropolitan areas in Panel B, and even after including additional covariates in the estimation equation (columns (2) and (3)). Results shown in column (2) of Panel B show that one additional unit of PM_2.5_ increases the mortality rate associated with the COVID-19 disease by 1.5 percent in municipalities belonging to metropolitan areas. This result is consistent with Fig 2, discussed earlier. This relationship increases in magnitude once we control for country-specific fixed effects, in column (3). The estimated incidence rate ratio in this column suggests that one additional microgram per cubic meter of PM_2.5_ increases the COVID-19 mortality rate by 2.7 percent for those municipalities in metropolitan areas.

Regarding municipalities in non-metropolitan areas, the results in Panel C show no statistically significant association between long-term average PM2.5 concentrations and mortality rate due to the coronavirus. Taken together, these results suggests that the likelihood of an increased risk of dying from COVID-19 due to long-term exposure to ambient air pollution is significant only in metropolitan areas of Latin America.

We provide two possible explanations for the differences found between metropolitan and non-metropolitan areas. On the one hand, in urbanized municipalities, people are exposed to high spikes of air pollution in periods of high traffic demand during commuting time to work or school. Being trapped in traffic congestion increases the time of pollution exposure and thus the health risks. On the other hand, recent research has emphasized the importance of particulate matter composition beyond the mass concentration, because pollutant composition may be associated with the acute and chronic health effects of air pollution. The composition of particulate matter may include hazardous materials for health such as heavy metals. For the case of Europe, for example, it has been found that urban sites not only exhibit higher mass concentration of particulate matter but also higher oxidative potential per unit of mass concentration than rural sites [52]. The higher the oxidative potential of particulate matter, the higher the toxicity, because the pollutant can generate reactive oxygen species that are harmful to cells [53]. This highlights that exposure to particulate matter in metropolitan areas may be more toxic than in non-metropolitan areas.

### Country-specific results

The link between long-term PM2.5 air pollution and COVID-19 deaths in each country is depicted in Table 4. Panel I shows results for Brazil, Panel II for Chile, and Panel III and IV for Colombia and Mexico, respectively. Columns (1) to (3) follow the same structure as in Table 3. This time, however, we add column (4), which depicts the results of estimating Eq (2) using the richer set of covariates as defined in the data section. For each country, we present results for all municipalities in Panel A, and municipalities in metropolitan and non-metropolitan areas in Panels B and C.

**Table 4:** Long-term average PM_2.5_ exposure and COVID-19 mortality rate by country.

	(1)	(2)	(3)	(4)
**Panel I. Brazil**				
*Panel A*. *All Municipalities*				
PM2.5	1.054***	1.023**	1.029**	1.020
	[1.030, 1.078]	[1.004, 1.041]	[1.001, 1.058]	[0.991, 1.051]
Obs.	5,546	5,514	5,514	5,513
*Panel B*. *Metropolitan Areas*				
PM2.5	1.045***	1.025**	1.057***	1.050***
	[1.019, 1.072]	[1.002, 1.047]	[1.023, 1.091]	[1.018, 1.084]
Obs.	1,400	1,396	1,396	1,395
*Panel C*. *Non-Metropolitan Areas*				
PM2.5	1.025**	1.010	0.971**	0.964**
	[1.006, 1.044]	[0.993, 1.027]	[0.945, 0.997]	[0.936, 0.993]
Obs.	4,146	4,118	4,118	4,118
**Panel II. Chile**				
*Panel A*. *All Municipalities*				
PM2.5	1.097***	1.100***	1.070***	1.060***
	[1.083, 1.111]	[1.077, 1.125]	[1.018, 1.124]	[1.034, 1.086]
Obs.	346	324	324	321
*Panel B*. *Metropolitan Areas*				
PM2.5	1.095***	1.088***	1.022	1.026**
	[1.078, 1.112]	[1.071, 1.106]	[0.988, 1.057]	[1.002, 1.050]
Obs.	64	64	64	62
*Panel C*. *Non-Metropolitan Areas*				
PM2.5	1.099***	1.108***	1.105***	1.086***
	[1.048, 1.152]	[1.062, 1.155]	[1.039, 1.175]	[1.043, 1.131]
Obs.	281	260	260	259
**Panel III. Colombia**				
*Panel A*. *All Municipalities*				
PM2.5	1.006	1.003	1.003	0.997
	[0.983, 1.030]	[0.989, 1.016]	[0.986, 1.020]	[0.978, 1.015]
Obs.	1,119	1,100	1,100	924
*Panel B*. *Metropolitan Areas*				
PM2.5	1.003	1.009	1.019***	1.014***
	[0.991, 1.014]	[0.998, 1.020]	[1.006, 1.032]	[1.012, 1.015]
Obs.	22	22	22	22
*Panel C*. *Non-Metropolitan Areas*				
PM2.5	1.001	0.994	1.008	0.999
	[0.977, 1.027]	[0.981, 1.007]	[0.984, 1.032]	[0.976, 1.024]
Obs.	1,097	1,078	1,078	902
**Panel IV. Mexico** *Panel A*. *All Municipalities*				
*Panel A*. *All Municipalities*				
PM2.5	1.024*	0.991	0.988	0.988
	[0.997, 1.052]	[0.978, 1.005]	[0.959, 1.017]	[0.966, 1.012]
Obs.	2,240	2,240	2,240	2,240
*Panel B*. *Metropolitan Areas*				
PM2.5	1.042***	1.026***	1.020**	1.024***
	[1.016, 1.068]	[1.023, 1.029]	[1.003, 1.038]	[1.006, 1.042]
Obs.	104	104	104	104
*Panel C*. *Non-Metropolitan Areas*				
PM2.5	0.977	1.002	1.004	0.997
	[0.950, 1.006]	[0.986, 1.018]	[0.977, 1.032]	[0.971, 1.023]
Obs.	2,136	2,136	2,136	2,136
Common-Set of Controls		*×*	*×*	
Richer-Set of Controls				*×*
State Fixed Effects			*×*	*×*

**Notes:** This table shows regression estimates of COVID-19 mortality rate on annual PM2.5 concentrations averaged from 2000 to 2018. Estimates as incidence rate ratios from Poisson regressions offsetting by population and clustering standard errors at the state level. Results for Brazil exclude Brasilia. Results in columns (3) and (4) of Panel III (B) include an Andean-fixed effect instead of state-fixed effects. Brackets show 95% confidence intervals. Significance levels: *p < 0.10, **p < 0.05, ***p < 0.01.

#### Brazil

The results for Brazil in Panel I of Table 4 show a positive relationship between long-term exposure to PM2.5 pollution and COVID-19 mortality rates, which is in line with the results presented in Table 3 for the four Latin American countries as a whole. In particular, the results in column (1) of Panel A using the full sample of Brazilian municipalities show that one additional unit of PM2.5 is significantly associated with a 5.1 percent increase in mortality rate due to COVID-19. This result remains statistically significant, but it decreases in magnitude once we consider other factors that may affect COVID-19 deaths, as observed in columns (2) and (3). When we control for the entire set of potentially related factors available for Brazil that may affect our outcome variable, the result from column (4) shows that an additional unit of PM2.5 is associated with a 2 percent increase in COVID-19 mortality rates. However, this result is no longer statistically significant.

The positive association between long-term pollution and COVID-19 mortality found for all Brazilian municipalities holds invariably when we consider only those municipalities within metropolitan areas in Panel B. This time, however, this association remains statistically significant regardless of the number of additional covariates that we control for in our estimation equation. More specifically, results from our preferred specification in column (4) suggests that one additional microgram of PM_2.5_ pollution is associated with a 5 percent increase in mortality rate due to COVID-19. When contrasting this result with the one found for all metropolitan municipalities of Latin American in Table 3, we observe that the deadly COVID-19 effects of pollution exposure are, on average, more critical in metropolitan areas of Brazil than in similar municipalities of other countries of the region. In fact, 58 percent of the municipalities in metropolitan areas of Brazil that present long-term average PM_2.5_ concentrations above the World Health Organization’s air quality guideline are located within the state of Sao Paulo–the most highly urbanized region in Brazil, and home of the largest vehicle fleet in the country. To the extent that long-term exposure to harmful concentrations of other pollutants, such as carbon monoxide (CO) or nitrogen oxides (NO_*X*_)—which in addition to PM_2.5_ are commonly emitted by combustion engines of light-duty vehicles—is exacerbating the risk of death from COVID-19, then the estimate shown in column (4) may reflect the overall effect of long-term exposure to a wider range of pollutants instead of capturing an effect that is exclusive to PM_2.5_ concentrations. Nonetheless, our estimate in column (4) gives us a first glimpse of the potential association between long-term exposure to harmful air pollution in general and the risk of death from COVID-19.

On the other hand, the results for non-metropolitan areas, presented in Panel C, seem to suggest a reduced risk of mortality from exposure to PM_2.5_ pollution. This is an unexpected finding which might be the result of something unique to Brazil. The country has been constantly suffering from a large number of wildfires that are increasingly affecting tropical forests, savannas, and wetlands, which are located in rural areas of the country (i.e., non- metropolitan areas). For instance, as shown in panel (a) of Fig 1, the highest PM_2.5_ levels are found in municipalities of the State of Mato Grosso, a state that concentrates almost 20 percent of all wildfire outbreaks detected in Brazil during 2000–2018 [54]. At the same time, and likely due to their low population density levels, the COVID-19 mortality rate in these municipalities was less than half the mortality rate in non-rural municipalities of the country in 2020. This suggests that our specification may be failing at capturing pollution dynamics that are specific to rural areas. Future research relating PM_2.5_ and COVID-19 fatalities could explore further the different sources of air pollution across rural and non-rural areas, which would better guide actions for pollution abatement.

#### Chile

The positive relationship between long-term PM_2.5_ exposure and COVID-19 fatalities is also observed in the results for Chile, presented in Panel II. The findings shown in Panel A indicate that one additional unit of PM_2.5_ is associated with an increase in COVID-19 mortality rate in the order of 6 to 10 percent (columns (1)-(4)). In our preferred specification using the richer set of controls (column (4)), we observe that one additional unit of PM_2.5_ is associated with a 6 percent increase in the mortality rate due to COVID-19.

This relationship is found positive and statistically significant for metropolitan areas as well, although it decreases in magnitude to a 2.6 percent in our preferred specification in column (4). Instead, for municipalities in non-metropolitan areas, we observe that this positive relationship becomes larger in magnitude and highly statistically significant. The results presented in column (4) of Panel C show that one additional unit of fine particulate matter is associated with an 8.6 percent increase in COVID-19 mortality rate. This finding is important because, unlike what is observed for other countries in the region, the results for non-metropolitan municipalities of Chile suggest that long-term exposure to fine particulate matter may be an important risk factor of dying from COVID-19 in municipalities that do not necessarily belong to highly urbanized areas. A plausible explanation for this result could relate to the different sources of PM_2.5_ emissions. Non-metropolitan municipalities of Chile are exposed to important sources of air pollution as well, which may differ from what is commonly known as the main sources of air pollution in metropolitan areas. For instance, mining activities and coal power generation is an important source of air pollution in non- metropolitan municipalities of northern Chile [55, 56]. Similarly, residential wood burning for heating is the main source of PM_2.5_ in non-metropolitan municipalities of south-central and south Chile [57]. Additionally, and unlike most countries in Latin America, Chile faces low temperatures during the winter period, which makes it more likely for the population of municipalities in non-metropolitan areas to be highly exposed to wood burning air pollution, at the same time that colder, and dry temperatures, constitute an additional risk factor for SARS-CoV-2 infection [53]. This situation could particularly exacerbate the risk of death due to critical exposure to air pollution in Chilean municipalities that do not necessarily belong to metropolitan areas, relative to non-metropolitan municipalities of more tropical countries such as Brazil or Colombia. Unfortunately, our data prevents us from testing this hypothesis, which remains an open avenue for future research.

#### Colombia

The results for Colombia in Panel III hold consistently with the results previously observed for the whole region in Table 3. Overall, we observe a positive relationship between long-term PM_2.5_ exposure and the risk of death due to COVID-19 across all municipalities. Yet, these results lack statistical significance. Notwithstanding, when we restrict the sample to municipalities that are located in metropolitan areas only, we observe a positive and highly significant association, which is in line with the results for Brazil and Chile. In particular, using the common-set of covariates in column (3), we observe that one additional unit of PM_2.5_ is associated with a 1.9 percent increase in the risk of death due to COVID-19. This result has a similar order of magnitude after taking into account additional factors that may also affect the mortality rate. In column (4), we observe an association of a 1.4 percent increase in the risk of COVID-19 death due to one extra unit of PM_2.5_ concentration, which is lower than the estimated average for the four countries in Latin America. It is important to highlight that municipalities of metropolitan areas in Colombia are urban concentrations that include Bogota, Medellin–among others–with high levels of mobile source air pollution as in the case of Brazil. Pollution levels in these highly urbanized areas have induced authorities in Colombia to yearly announce PM_2.5_-based air quality warnings to reduce exposure during periods of critical air pollution. In the case of non-metropolitan areas, however, we observe no significant relationship between PM_2.5_ and COVID-19 fatalities.

#### Mexico

Finally, the results in Panel IV of Table 4 for Mexican municipalities tell a consonant story regarding long-term exposure to air pollution as a risk factor for COVID-19 mortality, particularly in metropolitan areas. Starting with all municipalities in Panel A, we observe a positive and statistically significant relationship of 2.4 percent in column (1). Yet, this significance is lost once we include stronger controls in our estimation equations.

Unsurprisingly, when we look at municipalities that are located in metropolitan areas (Panel B) we find positive and statistically strong relationships. The findings from our preferred specification, in column (4), suggest that the risk of death due to a coronavirus infection increases by 2.4 percent for an additional unit of exposure to fine particulate matter. The magnitude of this effect is similar to the results found for metropolitan municipalities in Chile (in Panel II) and the average effect found for all metropolitan municipalities in Panel B of Table 3.

### Robustness checks

The previous results consistently show a positive and statistically significant association between long-term average PM_2.5_ exposure and COVID-19 mortality rate in metropolitan municipalities. Thus far, our definition of long-term exposure considers risk exposure to PM_2.5_ pollution records averaged over 19 years, from 2000 to 2018. While this has been the standard for historical or long-term PM_2.5_ pollution concentrations in the related literature (e.g., [17, 21]), it is important to notice that policy-makers of several of the metropolitan areas under analysis undertook multiple actions and tightened environmental standards to curb air pollution concentrations during this period of time [54–60]. Hence, one possibility is that, as a result of these many efforts, or simply because of more awareness, exposure to fine particulate matter concentrations may have been lower during the more recent years. This would cause our previous results to overstate the risk of exposure to air pollution as a factor in the probability of dying from COVID-19. To account for this, we estimate Eqs (1) and (2) using PM_2.5_ concentrations measured between 2010 and 2018 rather than since 2000. Doing this also helps us to deal with the possibility that people may have moved across cities during such a long period. This alternative test shows that our results remain unchanged when we use a more contemporaneous definition of long-term PM_2.5_ pollution exposure (see S2 Table for the results for the pooled-sample of countries and S3 Table for the country-specific results).

In addition to a redefinition of our long-term pollution exposure measure, we also consider a distinction between municipalities that, in our sample, have been historically above or below the WHO air quality guideline of annual fine particulate matter concentrations. We do so as to classify cities with critical levels of long-term exposure to air pollution in a reliable and exogenous manner, and to explore whether our findings hold exclusively for these municipalities. To that end, we create an indicator variable that takes the value of one for municipalities with long-term average PM_2.5_ concentrations equal or above 10 *μ*g/m^3^, and zero for municipalities below the guideline. By adding this indicator interacted with PM_2.5_ to our estimations, we obtain an incidence rate ratio that is specific to cities above and below this guideline, allowing us to better compare the relationship between air pollution and COVID-19 mortality rate across cities with and without exposure to high levels of air pollution.

The results of this additional specification are shown in S4 Table. For all municipalities in Panel A, we observe no statistically significant relationship between PM_2.5_ and COVID-19 mortality, not even among municipalities with high levels of exposure to PM_2.5_. Notwithstanding, when we consider only municipalities in metropolitan areas (Panel B), we observe that consistently throughout all three different specifications, there is a positive and significant association between PM_2.5_ exposure and COVID-19 fatalities, which is specific to municipalities with pollution records historically above the WHO’s air quality standard. We take the results in S4 Table as reassurance that long-term PM_2.5_ exposure constitutes a notable risk factor in the probability of dying from COVID-19 in Latin American cities exposed to high levels of air pollution.

We also test the robustness of our estimator by re-estimating Eqs (1) and (2) with a NBMLE. The results are depicted in S5 and S6 Tables, respectively. We observe that the results are vastly similar when pooling countries in S5 Table. We also obtain similar results when estimating country-specific results in S6 Table, although we lose some precision.

## Discussion

Our main result suggests that one additional unit of PM_2.5_ pollution is statistically associated with a 2.7 percent higher risk of dying from COVID-19 in metropolitan municipalities of Brazil, Chile, Colombia, and Mexico. This result is robust to several alternative specifications. Further analysis shows that this association holds only for municipalities of metropolitan areas that exceed WHO air quality guidelines for annual concentrations. This would suggest that the adverse health effects of exposure to PM_2.5_ on COVID-19 mortality are concentrated in urban areas that are exposed to high levels of air pollution over a long period of time.

Although not directly comparable, our findings are in line with previous estimates for other developing countries, such as those in [42], which find that one percent increase in long-term exposure to PM_2.5_ (*≈* 2.46 *μ*g/m^3^) leads to a 0.027 percent- age point increase in the mortality rate in India. For the case of Mexico, our results are slightly lower in magnitude relative to the estimates by [17], which use individual-level data for estimating the link between pollution exposure and COVID-19 mortality in the metropolitan area of Mexico City (MCMA). Nonetheless, in addition to those in the MCMA, our set of metropolitan municipalities in Mexico also includes municipalities in the Guadalajara’s and Monterrey’s conurbations. Given that long-term average PM_2.5_ concentrations recorded in these two metropolitan areas are roughly half the average concentrations observed for the MCMA, it is reasonable to expect that estimates that include these municipalities will be attenuated towards zero relative to the findings for the MCMA alone. Finally, our results for Colombia are different from those of [44], which find no statistically significant association between long-term exposure to PM_2.5_ pollution and COVID-19 mortality. While our data and methods differ from the ones in [44], it is highly plausible that our results differ due to the window of time for the mortality data: while [44] look at COVID-19 mortality up to July 17th, 2020, we use information for the whole year. Analysis of mortality data shows that roughly 50 percent of the Colombian municipalities reported their first COVID-19 case in August 2020 or later. That is, most of the COVID-19 mortality for year 2020 in Colombia is not captured by [44].

On the other hand, our estimates are smaller relative to other studies looking at this relationship in the context of developed countries. For instance [18], find that an additional unit of long-term exposure to PM_2.5_ is associated with a 9 percent higher COVID-19-related mortality in northern Italy [19], find a 13.6 percent for the Netherlands, and [21] find an 8 percent for the United States. Our results may seem conservative relative to these studies as we pool together information from several countries, each one of them with distinct air pollution mitigation policies and varying strategies to control the pandemic. In our country-specific results, however, we obtain associations that are similar in magnitude to that of these previous findings, such as in the case of metropolitan municipalities of Brazil or non-metropolitan municipalities of Chile.

Our study has some limitations due to the available data characteristics, which suggests caution when interpreting our results. First, we use municipality-level data. While informative, our approach may obscure individual-level actions taken to avoid coronavirus contagion and (or) mitigate exposure to air pollution. Future research could delve deeper into the behavioral aspect of this relationship. At the same time, our study is an ecological study, and thus, it may be prone to suffer from an ecological fallacy. Although it is possible that, for instance, our long-term average air pollution exposure may not necessarily be representative of individuals’ lifetime air pollution exposure, it is likely a good indicator of the central tendency of that distribution. Moreover, several scholars have highlighted these countries’ low internal migration rates [61, 62]. This minimizes fears over the possibility that our long-term pollution exposure measure may not be such an accurate picture of prolonged exposure to this hazard. Furthermore, previous studies that have used individual-level data instead document a similar relationship between long-term air pollution exposure and the individual likelihood of experiencing a fatal COVID- 19 related outcome for the case of Mexico [17]. Mexico’s population is relatively similar to other Latin American countries under analysis here, and thus, there are no reasons to expect that our results would not hold at the individual level.

An additional limitation relates to our homogeneous measure of pollution concentration within municipalities. If socioeconomically vulnerable individuals are less able to sort themselves towards low-pollution municipalities based on their preferences for air quality (or factors that closely correlate with it), and at the same time, they are more prone to develop a critical COVID-19 illness with deadly outcomes, then our results may be upward biased. Exploiting variation in pollution exposure within municipalities as well as exploring highly spatially resolved air pollution measures remain as a potential extension of our work. Finally, one possibility is that the confirmed number of COVID-19 fatalities used in this study will be underestimating the true death toll from the pandemic in the region. Although that emerges, unfortunately, as a plausible scenario, our results can still be considered a lower bound of the true association between long-lasting air pollution and the COVID-19 mortality rate. Even so, among the four countries under analysis, Chile appears to be the country with the lower disparity between excess deaths and deaths officially attributed to COVID-19 [63]. Consistent with our main result, we estimate that long-term air pollution exposure is associated with a 2.6% increase in the COVID-19 mortality rate in this country. Multiple other extensions of this paper are plausible. While our results provide strong evidence of a positive statistical association between long-term air pollution exposure and COVID-19 mortality rates in Latin America, they do not indicate causation. The focus on causal identification of this link, as well as an in-depth consideration of possible confounding effects and other sources of endogeneity that may exist behind it, remain as open avenues for future research. Other extensions to this paper could look at the relationship between COVID-19 deaths and air pollutants other than PM2.5, to better grasp the role of air pollution exposure as a risk factor in the COVID-19 mortality rate across non-metropolitan areas of Latin America. For instance, measures of other air pollutants could offer an alternative to exploring our research question in areas with a significant presence of extractive industries, wood-burning air pollution, or increasing wildfires, as in the case of non-metropolitan municipalities of Chile and Brazil, respectively. Likewise, a more comprehensive model of air pollution that takes into account meteorological variables could capture potential atmospheric dynamics that may affect air pollution concentrations. Finally, understanding to which extent long-term exposure to air pollution detrimentally correlates with income inequality and poverty is crucial for adequate policy design in Latin America. Future research could explore whether the harmful effects of long-term air pollution exposure on COVID-19 fatalities change in magnitude across different segments of the income distribution in these countries.

## Conclusions

The ongoing pandemic caused by the spread of the novel coronavirus has resulted in more than 4 million deaths worldwide. More than 30 percent of these fatalities have taken place in Latin America, one of the regions hardest hit by this pandemic. Latin America is characterized by high and long-standing levels of poverty and inequality, limited healthcare infrastructure and insufficient public health systems, scarce capability to follow quarantine and social distancing measures, and deficient access to social safety nets, which have all exacerbated the COVID-19 crisis in this region. In this paper, we examine the role of an additional factor fueling this crisis, long-term exposure to harmful levels of air pollution.

Our results show a positive relationship between long-term exposure to air pollution and COVID-19-related deaths in four countries that have been highly affected by the pandemic: Brazil, Chile, Colombia, and Mexico. This set of countries comprises a wide range of economies in Latin America, which offers variation in population size, geographic characteristics, and responses to the pandemic. Therefore, we believe that findings from this research are relevant for informing public health and environmental policy in the whole region.

Our results provide compelling evidence that channeling additional health care capacity and resources to the most polluted areas could mitigate some of the adverse health effects of COVID-19. Moreover, our findings support the need for strengthening environmental policies aimed at reducing ambient air pollution in Latin America, which could alleviate some of the devastating and unequal effects of the coronavirus pandemic and, arguably, other pandemics that might arise in the future. Finally, while trying to control the COVID-19 pandemic and its aftermath, Latin American countries may be tempted to lessen environmental regulations to foster economic activity [64]. Our findings show that doing so could not only be detrimental for the control of the current pandemic, it could also increase the negative effects of future disease outbreaks.

## Supporting information

S1 Appendix Data description and sources. (PDF)

S1 Fig 2019 daily PM_2.5_ concentrations in major metropolitan cities of Latin America. (PDF)

S1 Table Descriptive statistics on 2010–2018 average fine particulate matter (PM_2.5_) concentrations. (PDF)

S2 Table 2010–2018 average PM_2.5_ exposure and COVID-19 mortality rate in Latin American municipalities. (PDF)

S3 Table 2010–2018 average PM_2.5_ exposure and COVID-19 mortality rate by country. (PDF)

S4 Table Long-term average PM_2.5_ exposure and COVID-19 mortality rate in Latin American municipalities above and below the World Health Organization annual air quality guideline. (PDF)

S5 Table Long-term average PM_2.5_ exposure and COVID-19 mortality rate in Latin American municipalities, negative binomial. (PDF)

S6 Table Long-term average PM_2.5_ exposure and COVID-19 mortality rate by country, negative binomial. (PDF)
