# Supplementary material for: Association between long-term air pollution exposure and COVID-19 mortality in Latin America
Source: PLoS One. 2023 Jan 17;18(1):e0280355. doi: 10.1371/journal.pone.0280355 (PMC9844883; doi:10.1371/journal.pone.0280355)
Supplement: S1 Appendix — (PDF) [file pone.0280355.s001.pdf]

## S1 Appendix. Data description and sources

**Definition of Metropolitan Areas.** In Mexico, metropolitan areas are defined by the Ministry of Agrarian, Land and Urban Development (SEDATU), the National Population Council (CONAPO), and the National Institute of Statistics and Geography (INEGI); for this analysis we focus on metropolitan areas of at least 5 million inhabitants. In Chile, metropolitan areas are defined by the National Institute of Statistics (INE), while in Brazil by the Institute of Geography and Statistics (IBGE). In Colombia, however, there is no official classification of metropolitan areas, and thus, we use the functional urban area concept developed by the OECD (2020).

**Table A1. Full List of Covariates**

| | Brazil | Chile | Colombia | Mexico |
|---|---|---|---|---|
| **Panel A. Common-set:** | | | | |
| Number of days since first COVID-19 case | × | × | × | × |
| Number of hospital beds | × | × | × | × |
| Proportion of people between 15-44 years old | × | × | × | × |
| Proportion of people between 45-64 years old | × | × | × | × |
| Proportion of people 65 years old and older | × | × | × | × |
| Proportion of adults with less than high school | × | × | × | × |
| Proportion of rural population | × | × | × | × |
| Population density | × | × | × | × |
| **Panel B. Richer-set:** | | | | |
| Gini coefficient | | × | | |
| Median household income | × | × | | |
| Number of days since first at-home order | | × | | |
| Poverty rate or index | × | × | × | |
| Proportion of adults with less than elementary school | | × | | |
| Proportion of households owning a house | × | × | | |
| Proportion of native or minority population | × | × | | × |
| Proportion of population of African descent | × | | × | × |
| Proportion of workers in the mining industry | | × | | |
| Proportion of households with low overcrowding | | × | | |
| Proportion of households with medium overcrowding | | × | | |
| Proportion of households with high overcrowding | | × | | |
| Proportion of people with public health insurance | | × | | |
| Proportion of people without healthcare access | | | | × |
| Proportion of people with cardiovascular diseases | × | | | |
| Proportion of people with respiratory diseases | × | × | × | |
| Proportion of people with hypertension | | × | × | |
| Proportion of people with diabetes (type II) | × | × | × | |
| Proportion of people with obesity | | | × | |
| Number of diabetes deaths per 100,000 population | | | | × |
| Number of hypertension deaths per 100,000 population | | | | × |

Notes: The number of days since first COVID-19 case is the number of days since the first confirmed case in each country and December 31, 2020. The number of hospital beds is per 1,000 population. Population density is per $km^2$.

**Common-Set of Covariates.**

**Brazil.** Number of inhabitants, percentage of people for different age ranges (15-44, 45-64, and above 65), proportion of adults without high school and proportion of rural population come from the 2010 National Population Census (last available) collected by the Brazilian Institute of Geography and Statistics. Number of days since first COVID-19 case comes from the Ministry of Health. Number of hospital beds is the 2019 number of beds per 1,000 inhabitants computed at the state-level and obtained from the National Health Facilities Census (CNES), collected by the Ministry of Health.

**Chile.** Number of inhabitants, percentage of people for different age ranges (15-44, 45-64, and above 65), proportion of adults without high school and proportion of rural population come from the 2017 National Socioeconomic Characterization Survey (CASEN), a country-wide household survey with representation at the municipality-level. Number of days since first COVID-19 case comes from the Ministry of Health, through the Ministry of Science. This variable may be censored as the Ministry of Health publicly released municipality-level information starting on March 30, 2020 (first COVID-19 case in Chile was confirmed on March 3, 2020). Number of hospital beds is the 2019 number of beds per 1,000 inhabitants computed at the state-level and obtained from the Department of Health Statistics and Information (DEIS), part of the Ministry of Health.

**Colombia.** Number of inhabitants, percentage of people by age ranges (15-44, 45- 64, and above 65), and proportion of adults without high school, all come from the 2018 population census data reported by DANE. The number of hospital beds come from the Special Registration of Health Supply Service (REPS) during 2020. The number of days since first case is computed using the first day of symptoms. Proportion of inhabitants in rurality is calculated by CEDE for 2018.

**Mexico.** Number of inhabitants, percentage of people for different age ranges (15-44, 45-64, and above 65), and proportion of adults that did not finish high school were obtained from Mexico's 2020 Census of Population and Housing (INEGI, 2021). Number of beds per 1,000 inhabitants comes from INEGI for 2018. The number of days since first case is computed using the first day of symptoms according to data from the Ministry of Health. Proportion of rural population was calculated using information from the 2020 Census.

**<u>Richer-Set of Covariates</u>**.

**Brazil**. Socioeconomic and demographic variables come from the 2010 Population Cen- sus. Proportion of black and indigenous residents in the municipality also come from the census. Number of diabetes- and hypertension-related deaths per 100,000 population is obtained from the 2019 Mortality Information System, collected by the Ministry of Health. Rural and urban classification comes from the 2017's Classification and characterization of rural and urban spaces in Brazil, published by IBGE.

**Chile.** All socioeconomic and demographic variables come from the 2017 National Socioeconomic Characterization Survey (CASEN). The Gini coefficient uses households' total income. Median household income is in 2017 USD. Number of days since first at-home order considers the first state-level at-home order (i.e., the first at-home order within the state, regardless of the municipality). Overcrowding is defined as the ratio between household members and the total number of bedrooms available to them. Low overcrowding is defined as between 2,5 and 3,49 members per bedroom. Medium overcrowding as between 3,5 and 4,9 members, while high overcrowding is more than 5 members per bedroom. Proportion of people with certain disease is self-reported information.

**Colombia**. Percentages of multidimensional poverty and of ethnicity (black) come from the 2018 population census reported by DANE. The proportion of peopled with diabetes, hypertension, respiratory diseases and obesity come from the Integrated System of Social Protection (SISPRO) in 2018.

**Mexico.** Proportion of natives, African descents, and proportion of population speaking an indigenous language all come from the 2021 INEGI. The proportion of people without healthcare access is also gathered from 2021 INEGI. Number of diabetes- and hypertension-related deaths per 100,000 population is obtained from the 2018 INEGI.
