# Supplementary material for: Association between long-term air pollution exposure and COVID-19 mortality in Latin America
Source: PLoS One. 2023 Jan 17;18(1):e0280355. doi: 10.1371/journal.pone.0280355 (PMC9844883; doi:10.1371/journal.pone.0280355)
Supplement: S1 Fig — (PDF) [file pone.0280355.s002.pdf]

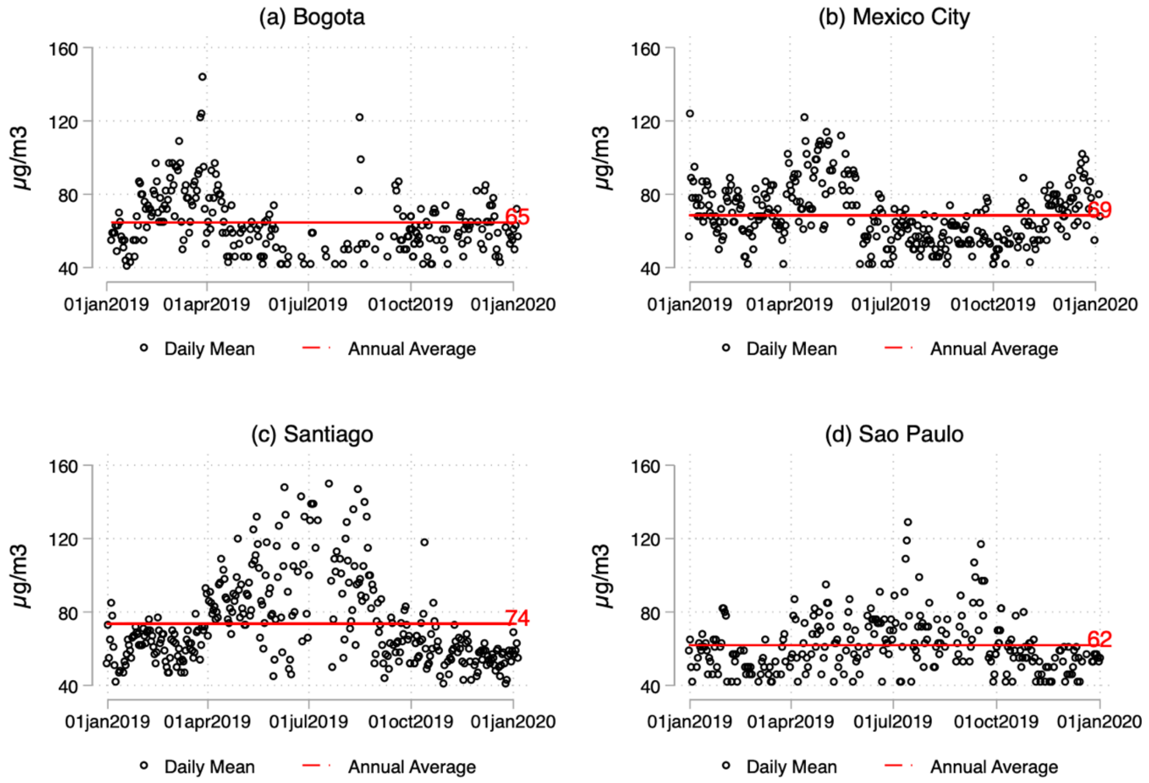

S1 Fig. 2019 Daily PM2.5 Concentrations in Major Metropolitan Cities of Latin America
This figure shows the 2019 daily median concentrations of ambient PM2.5 in four metropolitan cities in Latin America. Scatters are daily medians averaged across multiple stations in each city after trimming the top and bottom 5%. Horizontal lines are annual averages. Own elaboration using data from the Air Quality Data Open Platform, retrieved on February 2021 (aqicn.org/data-platform/covid19/).
