# Supplementary material for: Association between long-term air pollution exposure and COVID-19 mortality in Latin America
Source: PLoS One. 2023 Jan 17;18(1):e0280355. doi: 10.1371/journal.pone.0280355 (PMC9844883; doi:10.1371/journal.pone.0280355)
Supplement: S1 Table — (PDF) [file pone.0280355.s003.pdf]

S1 Table. Descriptive Statistics on 2010-2018 Average Fine Particulate Matter (PM2.5) Concentrations

Country	Mean	Std. Dev.	Min.	Max.	Obs.
Brazil	7.62	3.15	3.36	21.02	5,546
Chile	8.88	5.08	1.19	21.65	345
Colombia	23.45	6.17	9.23	37.27	1,119
Mexico	12.13	3.74	4.84	25.99	2,297

Notes: This table shows main descriptive statistics on 2010-2018 averages of annual PM2.5 pollution concentrations by country. Pollution is measured in $\mu g/m^3$. Observations are at the municipality level. Data correspond to long-term trends of fine particulate matter concentrations obtained from Hammer et al. (2020).
