# Supplementary material for: Association between long-term air pollution exposure and COVID-19 mortality in Latin America
Source: PLoS One. 2023 Jan 17;18(1):e0280355. doi: 10.1371/journal.pone.0280355 (PMC9844883; doi:10.1371/journal.pone.0280355)
Supplement: S2 Table — (PDF) [file pone.0280355.s004.pdf]

S2 Table. 2010-2018 Average PM2.5 Exposure and COVID-19 Mortality Rate in Latin American Municipalities

	(1)	(2)	(3)
Panel A. All Municipalities			
$PM_{2.5}$	1.015**	1.004	1.009
	[1.002, 1.029]	[0.994, 1.013]	[0.996, 1.022]
Obs.	9,235	9,235	9,235
Panel B. Metropolitan Areas			
$PM_{2.5}$	1.020***	1.013**	1.028***
	[1.006, 1.034]	[1.002, 1.024]	[1.015, 1.041]
Obs.	1,587	1,587	1,587
Panel C. Non-Metropolitan Areas			
$PM_{2.5}$	0.995	1.004	1.000
	[0.981, 1.009]	[0.994, 1.014]	[0.987, 1.014]
Obs.	7,648	7,648	7,648
Common-Set of Controls		x	x
Country Fixed Effects			x

Notes: This table shows regression estimates of COVID-19 mortality rate on annual PM2.5 concentrations averaged from 2010 to 2018. Results are estimates of incidence rates from Poisson regressions offsetting by population and clustering standard errors at the state level. Observations are municipalities. Common-set includes explanatory variables as defined above. Brackets show 95% confidence intervals. Significance levels: *p<0.10, **p<0.05, ***p<0.01
