# Supplementary material for: Association between long-term air pollution exposure and COVID-19 mortality in Latin America
Source: PLoS One. 2023 Jan 17;18(1):e0280355. doi: 10.1371/journal.pone.0280355 (PMC9844883; doi:10.1371/journal.pone.0280355)
Supplement: S3 Table — (PDF) [file pone.0280355.s005.pdf]

S3 Table. 2010-2018 Average PM2.5 Exposure and COVID-19 Mortality Rate by Country

	(1)	(2)	(3)	(4)
**Panel I. Brazil**				
Panel A. All Municipalities				
$PM_{2.5}$	1.054***	1.019**	1.031**	1.083***
	[1.030,1.079]	[1.002,1.038]	[1.004, 1.059]	[1.030, 1.138]
Obs.	5,546	5,514	5,514	5,513
Panel B. Metropolitan Areas				
$PM_{2.5}$	1.043***	1.021**	1.056***	1.052***
	[1.018,1.069]	[1.000,1.042]	[1.022, 1.092]	[1.016, 1.088]
Obs.	1,400	1,396	1,396	1,395
Panel C. Non-Metropolitan Areas				
$PM_{2.5}$	1.024**	1.006	0.967**	0.959**
	[1.004, 1.045]	[0.988, 1.026]	[0.937, 0.998]	[0.926, 0.994]
Obs.	4,146	4,118	4,118	4,118
**Panel II. Chile**				
Panel A. All Municipalities				
$PM_{2.5}$	1.095***	1.096***	1.068***	1.059***
	[1.081, 1.110]	[1.073, 1.120]	[1.019, 1.120]	[1.033, 1.086]
Obs.	345	324	324	321
Panel B. Metropolitan Areas				
$PM_{2.5}$	1.094***	1.087***	1.024	1.025**
	[1.077, 1.111]	[1.069, 1.104]	[0.987, 1.062]	[1.000, 1.051]
Obs.	64	64	64	62
Panel C. Non-Metropolitan Areas				
$PM_{2.5}$	1.089***	1.097***	1.097***	1.078***
	[1.041, 1.139]	[1.054, 1.142]	[1.034, 1.165]	[1.036, 1.121]
Obs.	281	260	260	259
**Panel III. Colombia**				
Panel A. All Municipalities				
$PM_{2.5}$	1.010	1.007	1.005	0.998
	[0.983, 1.038]	[0.994, 1.021]	[0.989, 1.021]	[0.981, 1.015]
Obs.	1,119	1,100	1,100	924
Panel B. Metropolitan Areas				
$PM_{2.5}$	1.006*	1.027**	1.038***	1.023***
	[1.000, 1.011]	[1.004, 1.050]	[1.013, 1.063]	[1.022, 1.023]
Obs.	22	22	22	22
Panel C. Non-Metropolitan Areas				
$PM_{2.5}$	1.004	0.996	1.011	1.001
	[0.976, 1.032]	[0.980, 1.011]	[0.988, 1.034]	[0.979, 1.024]
Obs.	1,097	1,078	1,078	902
Common-Set of Controls		×	×	
Richer-Set of Controls				×
State Fixed Effects			×	×

Continue on the next page

S3 Table. 2010-2018 Average PM2.5 Exposure and COVID-19 Mortality Rate by Country (cont.)

	(1)	(2)	(3)	(4)
**Panel IV. Mexico**				
Panel A. All Municipalities				
$PM_{2.5}$	1.028*	0.990	0.990	0.989
	[0.996, 1.061]	[0.974, 1.007]	[0.957, 1.023]	[0.962, 1.016]
Obs.	2,240	2,240	2,240	2,240
Panel B. Metropolitan Areas				
$PM_{2.5}$	1.049***	1.030***	1.022**	1.021**
	[1.019, 1.080]	[1.026, 1.034]	[1.001, 1.043]	[1.004, 1.039]
Obs.	104	104	104	104
Panel C. Non-Metropolitan Areas				
$PM_{2.5}$	0.974	1.002	1.007	0.999
	[0.941, 1.007]	[0.984, 1.021]	[0.975, 1.039]	[0.967, 1.032]
Obs.	2,136	2,136	2,136	2,136
Common-Set of Controls		×	×	
Richer-Set of Controls				×
State Fixed Effects			×	×

Notes: This table shows regression estimates of COVID-19 mortality rate on annual PM2.5 concentrations averaged from 2010 to 2018. Estimates as incidence rate ratios from Poisson regressions offsetting by population and clustering standard errors at the state level. Results for Brazil exclude Brasilia. Results in columns (3) and (4) of Panel III (B) include an Andean-fixed effect instead of state-fixed effects. Brackets show 95% confidence intervals. Significance levels: $*p < 0.10$, $**p < 0.05$, $***p < 0.01$.
