# Supplementary material for: Association between long-term air pollution exposure and COVID-19 mortality in Latin America
Source: PLoS One. 2023 Jan 17;18(1):e0280355. doi: 10.1371/journal.pone.0280355 (PMC9844883; doi:10.1371/journal.pone.0280355)
Supplement: S4 Table — (PDF) [file pone.0280355.s006.pdf]

S4 Table. Long-Term Average PM2.5 Exposure and COVID-19 Mortality Rate in Latin American Municipalities Above and Below the World Health Organization Annual Air Quality Guideline

	(1)	(2)	(3)
Panel A. All Municipalities			
$PM_{2.5}$ × Above Annual Guideline	1.017**	1.005	1.008
	[1.000, 1.035]	[0.994, 1.015]	[0.995, 1.022]
$PM_{2.5}$ × Below Annual Guideline	1.023	1.006	1.009
	[0.980, 1.068]	[0.980, 1.032]	[0.983, 1.036]
Obs.	9,235	9,235	9,235
Panel B. Metropolitan Areas			
$PM_{2.5}$ × Above Annual Guideline	1.016*	1.011*	1.024***
	[1.000, 1.032]	[0.999, 1.024]	[1.011, 1.037]
$PM_{2.5}$ × Below Annual Guideline	1.001	1.000	1.014
	[0.959, 1.046]	[0.968, 1.033]	[0.985, 1.044]
Obs.	1,587	1,587	1,587
Panel C. Non-Metropolitan Areas			
$PM_{2.5}$ × Above Annual Guideline	1.006	1.011**	1.006
	[0.9923, 1.021]	[1.002, 1.021]	[0.993, 1.019]
$PM_{2.5}$ × Below Annual Guideline	1.033*	1.028***	1.019
	[0.996, 1.071]	[1.008, 1.049]	[0.995, 1.043]
Obs.	7,648	7,648	7,648
Common-Set of Controls		×	×
Country Fixed Effects			×

Notes: This table shows regression estimates of COVID-19 mortality rate on annual PM2.5 concentrations averaged from 2000 to 2018 by municipalities above and below the World Health Organization's Air Quality Guideline of 10 μg/m^3 of annual PM2.5 concentrations. Estimates as incidence rate ratios from Poisson regressions offsetting by population and clustering standard errors at the state level. Observations are municipalities. Common-set includes explanatory variables as defined above. Brackets show 95% confidence intervals. Significance levels: *p < 0.10, **p < 0.05, ***p < 0.01.
