# Supplementary material for: Association between long-term air pollution exposure and COVID-19 mortality in Latin America
Source: PLoS One. 2023 Jan 17;18(1):e0280355. doi: 10.1371/journal.pone.0280355 (PMC9844883; doi:10.1371/journal.pone.0280355)
Supplement: S5 Table — (PDF) [file pone.0280355.s007.pdf]

S5 Table. Long-Term Average PM2.5 Exposure and COVID-19 Mortality Rate in Latin American Municipalities, Negative Binomial

	(1)	(2)	(3)
Panel A. All Municipalities			
$PM_{2.5}$	1.000	1.012***	1.008
	[0.987,1.014]	[1.004,1.021]	[0.995,1.021]
Obs.	9,235	9,235	9,235
Panel B. Metropolitan Areas			
$PM_{2.5}$	1.037***	1.021***	1.021***
	[1.020,1.055]	[1.008,1.034]	[1.006,1.036]
Obs.	1,587	1,587	1,587
Panel C. Non-Metropolitan Areas			
$PM_{2.5}$	0.995	1.013***	1.008
	[0.983,1.007]	[1.004,1.023]	[0.994,1.022]
Obs.	7,648	7,648	7,648
Common-Set of Controls		×	×
Country Fixed Effects			×

Notes: This table shows regression estimates of COVID-19 mortality rates on annual PM2.5 concentrations averaged from 2000 to 2018. Estimates shown are incidence rate ratios from Negative Binomial regressions offsetting by population and clustering standard errors at the state level. Observations are municipali- ties. Common-set includes explanatory variables as defined above. Brackets show 95% confidence intervals. Significance levels: *p < 0.10, **p < 0.05, ***p < 0.01.
