# Supplementary material for: Association between long-term air pollution exposure and COVID-19 mortality in Latin America
Source: PLoS One. 2023 Jan 17;18(1):e0280355. doi: 10.1371/journal.pone.0280355 (PMC9844883; doi:10.1371/journal.pone.0280355)
Supplement: S6 Table — (PDF) [file pone.0280355.s008.pdf]

**S6 Table. Long-Term Average $PM_{2.5}$ Exposure and COVID-19 Mortality Rate by Country, Negative Binomial**

| | (1) | (2) | (3) | (4) |
|---|---|---|---|---|
| **Panel I. Brazil** | | | | |
| *Panel A. All Municipalities* | | | | |
| $PM_{2.5}$ | 1.031*** | 1.011 | 1.000 | 0.991 |
| | [1.011, 1.050] | [0.994, 1.028] | [0.975, 1.027] | [0.965, 1.017] |
| Obs. | 5,546 | 5,514 | 5,514 | 5,513 |
| *Panel B. Metropolitan Areas* | | | | |
| $PM_{2.5}$ | 1.041*** | 1.013 | 1.044* | 1.033 |
| | [1.023, 1.059] | [0.991, 1.035] | [0.999, 1.091] | [0.992, 1.075] |
| Obs. | 1,400 | 1,396 | 1,396 | 1,395 |
| *Panel C. Non-Metropolitan Areas* | | | | |
| $PM_{2.5}$ | 1.024** | 1.011 | 0.983 | 0.973** |
| | [1.003, 1.045] | [0.994, 1.028] | [0.959, 1.006] | [0.950, 0.996] |
| Obs. | 4,146 | 4,118 | 4,118 | 4,118 |
| **Panel II. Chile** | | | | |
| *Panel A. All Municipalities* | | | | |
| $PM_{2.5}$ | 1.107*** | 1.104*** | 1.053** | 1.061*** |
| | [1.087, 1.128] | [1.078, 1.131] | [1.010, 1.099] | [1.025, 1.098] |
| Obs. | 345 | 324 | 324 | 321 |
| *Panel B. Metropolitan Areas* | | | | |
| $PM_{2.5}$ | 1.098*** | 1.076*** | 1.002 | 1.008 |
| | [1.073, 1.124] | [1.060, 1.093] | [0.972, 1.033] | [0.985, 1.032] |
| Obs. | 64 | 64 | 64 | 62 |
| *Panel C. Non-Metropolitan Areas* | | | | |
| $PM_{2.5}$ | 1.113*** | 1.121*** | 1.070** | 1.077*** |
| | [1.069, 1.158] | [1.087, 1.157] | [1.010, 1.133] | [1.032, 1.125] |
| Obs. | 281 | 260 | 260 | 259 |
| **Panel III. Colombia** | | | | |
| *Panel A. All Municipalities* | | | | |
| $PM_{2.5}$ | 1.001 | 1.004 | 1.012 | 1.006 |
| | [0.983, 1.021] | [0.991, 1.017] | [0.991, 1.033] | [0.984, 1.028] |
| Obs. | 1,119 | 1,100 | 1,100 | 924 |
| *Panel B. Metropolitan Areas* | | | | |
| $PM_{2.5}$ | 1.009 | 1.020*** | 1.024*** | 1.020 |
| | [0.991, 1.027] | [1.011, 1.030] | [1.017, 1.031] | [0.995, 1.047] |
| Obs. | 22 | 22 | 22 | 22 |
| *Panel C. Non-Metropolitan Areas* | | | | |
| $PM_{2.5}$ | 1.002 | 1.003 | 1.012 | 1.006 |
| | [0.983, 1.022] | [0.990, 1.017] | [0.990, 1.035] | [0.983, 1.030] |
| Obs. | 1,097 | 1,078 | 1,078 | 902 |
| Common-Set of Controls | | × | × | |
| Richer-Set of Controls | | | | × |
| State Fixed Effects | | | × | × |

*Continue on the next page*

**S6 Table. Long-Term Average $PM_{2.5}$ Exposure and COVID-19 Mortality Rate by Country, Negative Binomial (cont.)**

| | (1) | (2) | (3) | (4) |
|---|---|---|---|---|
| **Panel IV. Mexico** | | | | |
| *Panel A. All Municipalities* | | | | |
| $PM_{2.5}$ | 1.018 | 0.998 | 1.003 | 1.000 |
| | [0.987, 1.050] | [0.973, 1.024] | [0.981, 1.025] | [0.985, 1.016] |
| Obs. | 2,240 | 2,240 | 2,240 | 2,240 |
| *Panel B. Metropolitan Areas* | | | | |
| $PM_{2.5}$ | 1.046*** | 1.030*** | 1.056*** | 1.066*** |
| | [1.015, 1.078] | [1.025, 1.035] | [1.021, 1.092] | [1.022, 1.113] |
| Obs. | 104 | 104 | 104 | 104 |
| *Panel C. Non-Metropolitan Areas* | | | | |
| $PM_{2.5}$ | 1.002 | 0.999 | 1.015 | 1.010 |
| | [0.966, 1.041] | [0.971, 1.028] | [0.990, 1.041] | [0.986, 1.035] |
| Obs. | 2,136 | 2,136 | 2,136 | 2,136 |
| Common-Set of Controls | | × | × | |
| Richer-Set of Controls | | | | × |
| State Fixed Effects | | | × | × |

**Notes:** This table shows regression estimates of COVID-19 mortality rate on annual PM2.5 concentrations averaged from 2000 to 2018. Estimates as incidence rate ratios from Negative Binomial regressions offsetting by population and clustering standard errors at the state level. Brazilian results exclude Brasilia. Results in columns (3) and (4) of Panel III (B) include an Andean-fixed effect instead of state-fixed effects. Brackets show 95% confidence intervals. *p < 0.10, **p < 0.05, ***p < 0.01.
